# Die perioperative Antibiotikaprophylaxe – Darstellung der aktuellen Versorgungsrealität in der Endoprothetik in Deutschland und Erstellung einer Standardarbeitsanweisung für die Primär‑, Revisions- und Tumorendoprothetik

**DOI:** 10.1007/s00132-025-04761-1

**Published:** 2026-01-12

**Authors:** Andre Lunz, Andreas Geisbüsch, Frederike Lund, Sabrina Klein, Tilman Walker, Burkhard Lehner

**Affiliations:** 1 Zentrum für Orthopädie, Unfallchirurgie und Paraplegiologie, Universitätsklinikum Heidelberg, Schlierbacher Landstr. 200A, 69118 Heidelberg, Deutschland; 2 Klinik für Anästhesiologie, Universitätsklinikum Heidelberg, Im Neuenheimer Feld 420, 69120 Heidelberg, Deutschland; 3 Zentrum für Infektiologie, Medizinische Mikrobiologie und Hygiene, Universitätsklinikum Heidelberg, Im Neuenheimer Feld 324, 69120 Heidelberg, Deutschland

**Keywords:** Perioperative Antibiotika Prophylaxe, Postoperative Wundinfektionen, Endoprothetik, Revisionsendoprothetik, Tumorprothese, Periprothetische Infektion, Perioperative antibiotic prophylaxis, Surgical site infection, Total joint arthroplasty, Revision total joint arthroplasty, Tumor prosthesis, Periprosthetic joint infection

## Abstract

**Hintergrund:**

Die perioperative Antibiotikaprophylaxe (PAP) stellt eine zentrale Maßnahme zur Prävention postoperativer Infektionen dar. Insbesondere in der Endoprothetik besitzt die Infektionsprävention eine herausragende Bedeutung aufgrund des Risikos der bakteriellen Biofilmbildung auf der Implantatoberfläche im Infektionsfall.

**Ziel der Arbeit:**

Aufarbeitung und Darstellung der PAP an deutschen Endoprothetikzentren der Maximalversorgung (EPZmax), Vergleich der Ergebnisse mit der aktuell gültigen AWMF-S3 Leitlinie sowie differenzierte Darstellung der aktuellen Literatur zur PAP in der Revisions- und Tumorendoprothetik.

**Material und Methoden:**

Im Jahr 2023 wurde eine bundesweite Umfrage zur PAP an 100 EPZmax-Kliniken durchgeführt. Ergänzend wurde eine umfassende Literatur- und Leitlinienrecherche durchgeführt und eine klinikinterne, interdisziplinäre Standardarbeitsanweisung (SAA) erstellt.

**Ergebnisse:**

Insgesamt nahmen 45 Kliniken an der Umfrage teil. Cephalosporine der 1. und 2. Generation werden zu 98 % in der Primär- und 95 % in der Revisionsendoprothetik verwendet. Die PAP erfolgt zu 98 % in der Primär- und 67 % in der Revisionsendoprothetik als Einmalgabe und wird in 95 % bzw. 77 % der Fälle innerhalb 30 min vor dem Hautschnitt gegeben. Die Evidenzgüte wird in der Primärendoprothetik als hoch, in der Revisionsendoprothetik als gering bis mäßig von den teilnehmenden Kliniken eingeschätzt.

**Diskussion:**

Die Umfrageergebnisse zeigen eine weitgehend leitlinienkonforme Umsetzung der PAP in der Primärendoprothetik, jedoch eine heterogene Situation in der Revisionsendoprothetik. Die von den Autoren interdisziplinär erstellte SAA basiert auf den Empfehlungen der aktuell gültigen AWMF-S3 Leitlinie zu Substanzwahl, Dosierung und Zeitpunkt der PAP-Gabe sowie zum differenzierten Vorgehen bei Vorliegen einer Penicillin-Allergie. Abweichend hiervon wird unter Berücksichtigung der verfügbaren Evidenz und der Empfehlungen internationaler Fachgremien eine verlängerte PAP für bis zu 24 h in der Revisions- und Tumorendoprothetik empfohlen, um der besonderen Risikokonstellation bei diesen Eingriffen (erhöhtes Infektionsrisiko, potenziell schwerwiegende Folgen bei Auftreten einer Infektion) Rechnung zu tragen.

**Graphic abstract:**

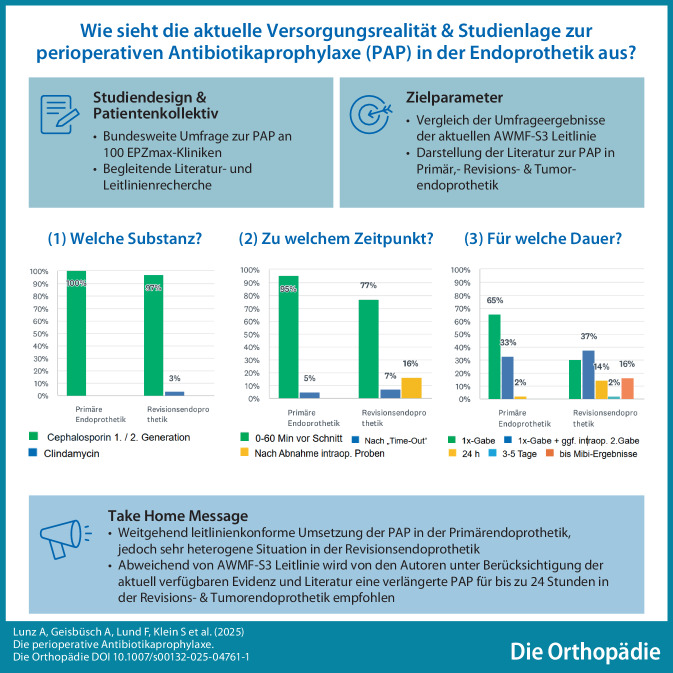

## Abstract (en)

**Background:**

Perioperative antibiotic prophylaxis (PAP) is one of the key measures for preventing surgical site infections (SSI). In total joint replacement (TJA), infection prevention is crucial due to the risk of bacterial biofilm formation in the event of infection.

**Objective:**

The study’s goal was to analyze the current clinical practise in German tertiary TJA centers (EPZmax), assess adherence to the currently valid German AWMF-S3 guideline, and present the contemporary literature on PAP in revision and tumor TJA.

**Materials and methods:**

In 2023, a nationwide survey on PAP practices was conducted among 100 EPZmax institutions. In addition, a comprehensive review of the relevant literature and guidelines was performed, and an interdisciplinary institutional standard operating procedure (SOP) was developed.

**Results:**

A total of 45 centers participated in the survey. First- and second-generation cephalosporins are used in 98% of primary and 95% of revision arthroplasties. In 98% of primary and 67% of revision procedures, PAP is administered as a single dose with 95% and 77% given within 30 minutes prior to incision, respectively. The evidence level for PAP was rated by the participating centers as high in primary and low to moderate in revision arthroplasty.

**Conclusion:**

The survey findings demonstrate guideline-conform implementation of PAP in primary TJA but reveal a heterogeneous situation in revision TJA. The interdisciplinary SOP written by the authors is based on the recommendations of the currently valid German guideline regarding the choice of antibiotic, dosing, and timing of PAP administration as well as on the differentiated approach in patients with a documented penicillin allergy. In deviation from the currently valid German guideline, and taking into account the available evidence and international expert consensus, an extended PAP for up to 24 hours is recommended for revision and tumor TJA, in order to adequately address the particular risk profile of these procedures (increased risk of infection and the potentially very severe consequences in the event of infection).

Die perioperative Antibiotikaprophylaxe (PAP) ist eine etablierte und evidenzbasierte Maßnahme zur Senkung postoperativer Infektionen. Ihre Wirksamkeit hängt entscheidend von der korrekten Umsetzung ab. Die aktuelle S3-Leitlinie der AWMF definiert einen evidenzbasierten Rahmen, berücksichtigt jedoch nicht die besonderen Konstellationen der Revisions- und Tumorendoprothetik, die mit einem signifikant erhöhten Infektionsrisiko und weitreichenden Konsequenzen beim Auftreten einer Infektion einhergehen. Bei solchen definierten Risikokonstellationen empfehlen die Autoren dieser Arbeit eine verlängerte Gabe der PAP für bis zu 24 h.

Postoperative Wundinfektionen („surgical site infections“, SSI) zählen zu den häufigsten nosokomialen Infektionen in Deutschland [24]. Sie führen zu einer erhöhten Morbidität und Mortalität, zu verlängerten stationären Verweildauern sowie zusätzlichen Kosten von schätzungsweise ca. 2 Mrd. Euro jährlich [6, 16, 22]. Ein zentraler Bestandteil der Maßnahmen zur Prävention postoperativer Infektionen ist die perioperative Antibiotikaprophylaxe (PAP). Zahlreiche randomisierte kontrollierte Studien (RCTs) und Metaanalysen belegen, dass eine korrekt durchgeführte PAP das Risiko für SSI signifikant reduziert [3, 14]. Zur Standardisierung werden regelmäßig nationale und internationale Empfehlungen publiziert und aktualisiert, in Deutschland zuletzt durch die S3-Leitlinie der Arbeitsgemeinschaft der Wissenschaftlichen Medizinischen Fachgesellschaften e. V. (AWMF) „Perioperative und periinterventionelle Antibiotikaprophylaxe“ [4]. Besonders schwerwiegend sind Infektionen nach operativen Eingriffen mit Implantation von Fremdmaterial (z. B. Osteosynthesen, Spondylodesen, Endoprothesen), da es hierbei zur bakteriellen Besiedelung und Biofilmbildung auf der Implantatoberfläche kommen kann, wodurch die Therapie erschwert wird. Im Endoprothesenregister Deutschland stellen periprothetische Infektionen („periprosthetic joint infections“, PJI) nach aseptischer Lockerung die zweithäufigste Revisionsursache dar; in der Revisionsendoprothetik sind sie mittlerweile sogar die häufigste Ursache [29]. Die hieraus resultierenden Konsequenzen – komplexe Revisionsoperationen, langwierige Behandlungsverläufe, hohe Behandlungskosten sowie hohe Morbidität und Mortalität – unterstreichen die Bedeutung einer konsequenten Infektionsprävention in der Endoprothetik. Der vorliegende Beitrag widmet sich daher neben den allgemeinen Empfehlungen zur PAP insbesondere den spezifischen Aspekten der PAP in der Primär‑, Revisions- und Tumorendoprothetik.

## Fragestellung

Vor dem Hintergrund der steigenden PJI-Inzidenz sowie deren hoher Relevanz für Morbidität, Mortalität und Gesundheitsökonomie kommt der adäquaten Durchführung der PAP eine zentrale Bedeutung zu. Trotz des Vorhandenseins nationaler und internationaler Leitlinien bestehen Hinweise auf eine heterogene Umsetzung in der klinischen Praxis. Zudem wird diskutiert, ob die derzeit gültigen allgemeinen Empfehlungen bestimmte operative Situationen (z. B. Revisions- und Tumorendoprothetik) ausreichend berücksichtigen.

Vor diesem Hintergrund ergeben sich folgende zentrale Fragestellungen:

1. Wie stellt sich die aktuelle Versorgungsrealität hinsichtlich der Umsetzung der PAP an Endoprothetikzentren der Maximalversorgung (EPZmax) in Deutschland dar?
2. Entspricht die Durchführung der PAP in der klinischen Routine den aktuell gültigen Empfehlungen/Leitlinien?
3. Wie stellt sich die aktuelle Studienlage zur PAP in der Revisions- und Tumorendoprothetik dar und wird das Risikoprofil dieser Operationen in der aktuellen AWMF-S3-Leitlinie ausreichend berücksichtigt?

## Studiendesign und Untersuchungsmethoden

Zur Beantwortung der ersten beiden Fragestellungen wurde im Jahr 2023 eine nationale Umfrage zur PAP in der Endoprothetik an 100 deutschen EPZmax-Kliniken durchgeführt. Bei der Auswahl wurden zunächst alle Universitäts- und berufsgenossenschaftlichen (BG) Kliniken sowie eine zufällige Auswahl an regionalen Krankenhäusern und Fachkliniken berücksichtigt. Einziges Einschlusskriterium war eine gültige Auszeichnung als EPZmax-Klinik sowie eine gültige E‑Mail-Adresse auf der klinikeigenen Homepage. Die anonyme Erhebung umfasste 18 Multiple-Choice-Fragen und wurde digital unter Verwendung der Software SurveyMonkey (Online Survey Tool; SurveyMonkey Inc., San Mateo, CA, USA) durchgeführt. Die deskriptive sowie statistische Auswertung der erhobenen Daten erfolgte mit Microsoft Excel (Version 2021; Microsoft Corporation, Redmond, WA, USA) und IBM SPSS Statistics (Version 29.0; IBM Corporation, Armonk, NY, USA).

Zur Beantwortung der dritten Fragestellung führten die Autoren dieser Arbeit eine umfassende, wenngleich nicht systematische Literaturrecherche zur PAP in der Primär‑, Revisions- und Tumorendoprothetik bei PubMed durch. Im Mittelpunkt standen aktuelle, vorzugsweise prospektive oder randomisierte Studien sowie Metaanalysen aus hochrangigen, internationalen und „peer-reviewed“ Fachzeitschriften. Auf dieser Grundlage sowie anhand der Empfehlungen der Langfassung der AWMF-S3 Leitlinie zur PAP wurde anschließend am universitären Standort der Autoren im interdisziplinären Konsens eine klinikinterne Standardarbeitsanweisung erarbeitet, die im vorliegenden Beitrag vorgestellt wird.

## Ergebnisse

**Abb. 1:**
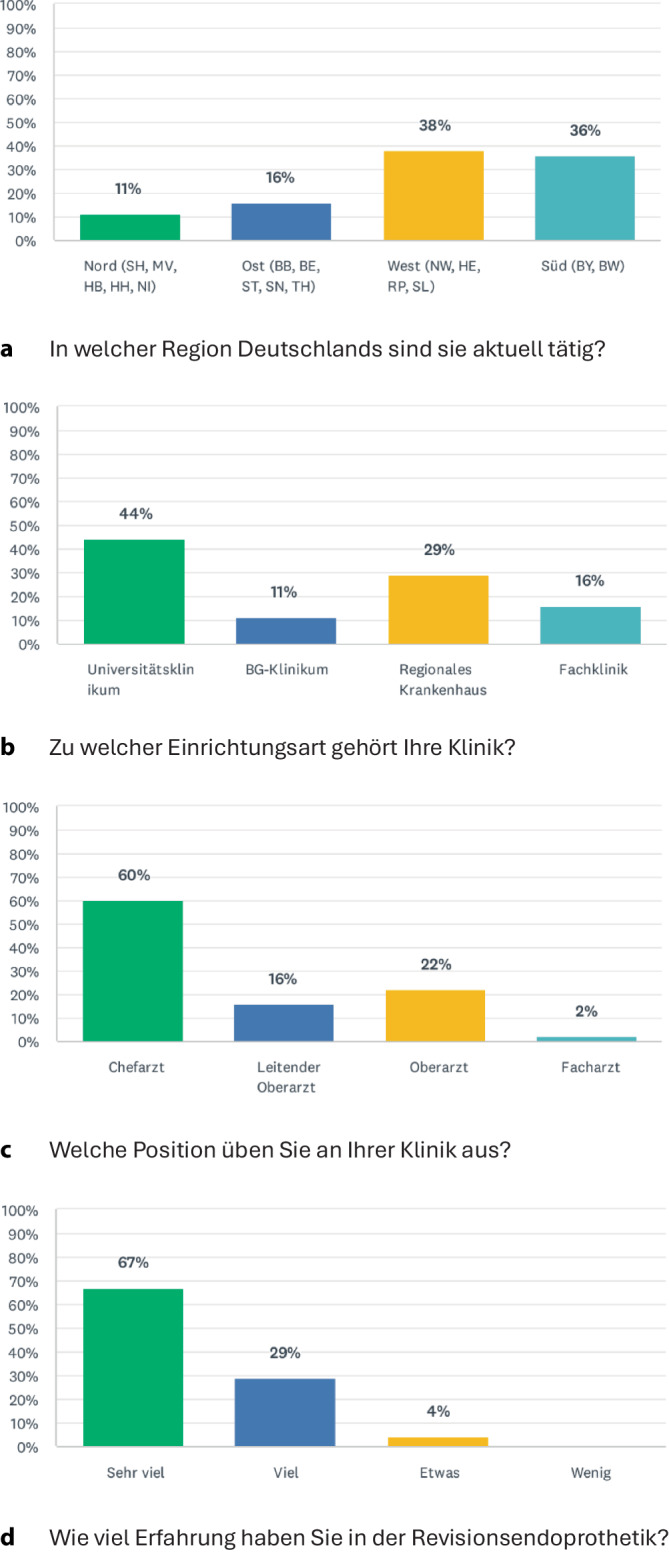
Allgemeiner Teil des Fragebogens mit Angaben über **a** Region, **b** Einrichtungsart, **c** Position und **d** Erfahrung der Interviewteilnehmer

Von den 100 angeschriebenen EPZmax-Kliniken nahmen 45 Einrichtungen (Rücklaufquote 45 %) an der Befragung teil und konnten in die Auswertung eingeschlossen werden (Abb. 1). Der größte Anteil der teilnehmenden Kliniken stammte aus Süd- (38 %) und Westdeutschland (36 %). Fast die Hälfte der beteiligten Einrichtungen waren Universitätsklinika (44 %), gefolgt von regionalen Krankenhäusern (29 %), Fach- (16 %) und BG-Kliniken (11 %). In 60 % der Fälle erfolgte die Beantwortung durch den Chefarzt, in 16 % durch den Leitenden Oberarzt und in 22 % durch einen Oberarzt der jeweiligen Abteilung. Bezüglich der Erfahrung im Bereich der Revisionsendoprothetik gaben 67 % der Befragten an, über „sehr viel“ und 29 % über „viel“ Erfahrung zu verfügen.

**Abb. 2:**
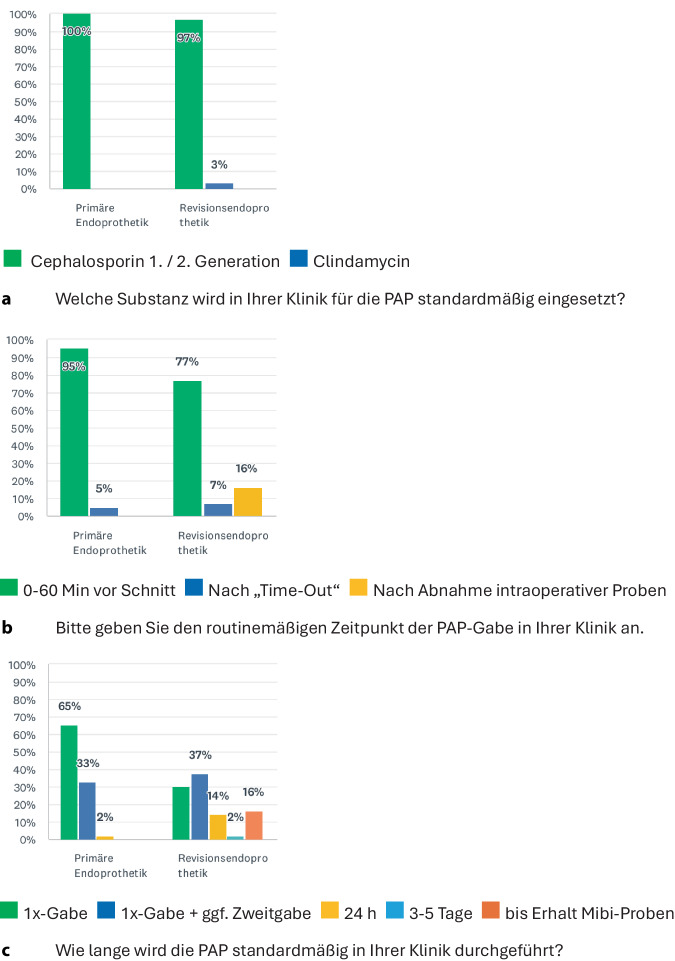
Spezifischer Teil des Fragebogens mit Angaben zur **a** Substanz, **b** Zeitpunkt der Gabe und **c** Dauer der perioperativen Antibiotikaprophylaxe (PAP)

Cephalosporine der ersten oder zweiten Generation werden in 98 % der Kliniken in der Primär- und in 95 % in der Revisionsendoprothetik routinemäßig eingesetzt. Die Applikation erfolgt zu 95 % in der Primär- und zu 77 % in der Revisionsendoprothetik ca. 30 min vor Hautschnitt. Die Antibiotikaprophylaxe wird als Einmalgabe zu 98 % in der Primär- und zu 67 % in der Revisionsendoprothetik angegeben. In 14 % der Kliniken wird in der Revisionsendoprothetik eine Mehrfachgabe (> 2 Gaben) am Operationstag durchgeführt, während weitere 16 % die Antibiotikagabe standardmäßig bis zum Vorliegen der mikrobiologischen Befunde fortsetzen (Abb. 2).

Die Evidenzgüte hinsichtlich des Zeitpunkts und der Dauer der Antibiotikaprophylaxe wurde für die Primärendoprothetik von etwa zwei Dritteln der Befragten als „hoch“ bewertet. In der Revisionsendoprothetik dagegen wurde die Evidenzgüte für Zeitpunkt und Dauer der Antibiotikagabe von jeder zweiten Klinik als „gering“ bis „mäßig“ eingestuft.

## Diskussion

Die PAP stellt eine der zentralen Maßnahmen zur Prävention postoperativer Infektionen dar. Ihre Wirksamkeit ist durch zahlreiche RCTs und Metaanalysen belegt. In einem systematischen Review konnte durch die Anwendung einer PAP eine relative Risikoreduktion von SSI um 81 % im Vergleich zu Placebo nachgewiesen werden [2]. Die pathophysiologische Grundlage dieser Effektivität liegt in der perioperativ temporär aufgehobenen Integrität der Haut- und Weichteilbarriere. Während dieses Zeitraums besteht ein erhöhtes Risiko einer mikrobiellen Kontamination des Operationsgebiets. Besonders bei Eingriffen mit Implantation von Fremdmaterial (z. B. Endoprothesen, Osteosynthesen oder Spondylodesen) ist die zur Auslösung einer Infektion notwendige Erregerlast signifikant reduziert. Klassische experimentelle Arbeiten von Elek u. Cohen et al. [17] sowie später von Gristina et al. [19] zeigten, dass perioperativ bereits eine um den Faktor 10.000 geringere Erregermenge im Bereich von Fremdmaterialien zur Infektion führen kann [17, 19]. Die PAP bietet einen effektiven Schutz in dieser vorübergehenden Phase der erhöhten Infektvulnerabilität. Entscheidend für die Wirksamkeit der PAP ist die korrekte Anwendung in Bezug auf Wirkstoffwahl, Dosierung, Applikationszeitpunkt und Dauer.

### Substanzwahl und Dosis der PAP

Eine optimale PAP sollte bakterizid wirken, nebenwirkungsarm und zugleich kosteneffizient sein und das zu erwartende Erregerspektrum gezielt abdecken. Darüber hinaus sind bei der Wahl des Antibiotikums zur PAP die lokale Resistenzsituation der Klinik sowie patientenspezifische Risikofaktoren zu berücksichtigen. Cephalosporine der ersten (Cefazolin) und zweiten Generation (Cefuroxim) gelten als Standardpräparate zur PAP. Cefazolin zeigt eine ausgezeichnete Wirksamkeit gegenüber grampositiven Erregern, insbesondere Staphylokokken, und besitzt ein begrenztes Wirkspektrum gegenüber gramnegativen Erregern [31]. Zudem ist das Risiko einer *Clostridioides-difficile-*Infektion unter Cefazolin im Vergleich zu Cefuroxim geringer [15]. Die klinische Überlegenheit von Cefazolin als PAP konnte durch mehrere groß angelegte Studien belegt werden. In der retrospektiven Analyse von Buchalter et al. [10] mit nahezu 11.500 primären Totalendoprothesen (TEP) war die PAP mit Cefazolin im Vergleich zu Nicht-Cephalosporin-Regimen (Vancomycin oder Clindamycin) mit einer signifikant niedrigeren 90-Tage-Inzidenz periprothetischer Infektionen (0,5 % vs. 1,0 %; *p* = 0,012) assoziiert. Über ähnliche Ergebnisse berichteten Carter et al. [12] in einer Registerstudie mit fast 290.000 primären TEPs, in der Cefazolin im Vergleich zu Nicht-Cefazolin-Regimen (alle anderen Antibiotika, inklusive Cefuroxim) mit einer signifikant niedrigeren PJI-Rate (0,5 % vs. 0,9 %; *p* < 0,001) verbunden war. Unter Berücksichtigung des Wirkspektrums, des Nebenwirkungsprofils, der Empfehlungen der AWMF-S3 Leitlinie sowie der aktuellen Evidenzlage werden 2 g Cefazolin i.v. als Mittel der 1. Wahl für die PAP empfohlen *(„hohe Evidenzgüte“)*.

Ein weiterer wichtiger Aspekt ist die gewichtsadaptierte Dosierung der PAP mit dem Ziel der Vermeidung unzureichender Plasmawirkspiegel. In der S3-Leitlinie wird auf Basis eines Expertenkonsenses eine Dosiserhöhung von u. a. Cefazolin auf 3 g ab einem Körpergewicht von 100–120 kg empfohlen. Die aktuelle Datenlage zur gewichtsadaptierten Dosierung der PAP und deren Bewertung in internationalen Leitlinien ist heterogen: Viele Studien zur PAP bei adipösen Patienten zeigen niedrigere Plasma- und Gewebekonzentrationen bei Standarddosierungen, jedoch konnte in nur wenigen Studien eine tatsächliche Senkung der Wundinfektionsrate durch den Einsatz gewichtsadaptierter höherer Dosierungen gezeigt werden [25]. In einer prospektiven, multizentrischen Studie mit fast 2000 primären TEPs konnten Badge et al. [5] nachweisen, dass eine gewichtsadaptierte Dosiserhöhung zu einer signifikanten Reduktion des postoperativen Infektionsrisikos führte. Einschränkend ist jedoch zu bemerken, dass in der Studie die Dosiserhöhung von 1 g bei < 120 kg KG auf 2 g bei > 120 kg KG untersucht wurde. Dennoch unterstreicht diese Studie, dass eine PAP-Unterdosierung mit einem signifikant erhöhten Infektionsrisiko einhergeht. Rondon et al. [25] konnten in ihrer retrospektiven Studie mit über 17.000 primären TEPs zeigen, dass adipöse Patienten mit einem Gewicht von über 120 kg, die eine Dosis < 3 g Cefazolin zur PAP erhalten haben, ein signifikant erhöhtes PJI-Risiko im Vergleich zu Patienten, die eine gewichtsadaptierte Dosierung der PAP erhielten, besitzen. In Einklang mit dem Expertenkonsens der aktuellen AWMF-S3 Leitlinie und im Sinne einer Risiko-Nutzen-Abwägung empfehlen die Autoren dieser Arbeit bei Patienten mit einem Körpergewicht über 100–120 kg die gewichtsadaptierte Dosierung von 3 g Cefazolin i.v. zur PAP bei allen endoprothetischen Eingriffen *(„moderate Evidenzgüte“ *bzw.* „Expertenmeinung“)*.

### Zeitpunkt der PAP

Alle internationalen Leitlinien empfehlen übereinstimmend die Applikation der PAP als Einmalgabe vor dem Hautschnitt, um zum Zeitpunkt der vorübergehenden iatrogenen Aufhebung der Hautbarriere bereits ausreichende Wirkspiegel im Blut und im Operationsgebiet sicherzustellen [4, 8]. Eine intraoperative Zweitgabe ist lediglich bei Überschreiten der doppelten Halbwertszeit des verwendeten Antibiotikums oder bei einem Blutverlust über 1–1,5 Litern empfohlen ([4],* „hohe Evidenzgüte“)*.

Auch in der Endoprothetik liegen robuste klinische Daten vor, die den Stellenwert der Gabe vor dem Hautschnitt verdeutlichen. In einer prospektiven multizentrischen Studie mit fast 4500 TEPs konnten Steinberg et al. [28] zeigen, dass das Risiko postoperativer Infektionen bei Applikation nach Hautschnitt im Vergleich zu einer präoperativen Gabe nahezu verdoppelt war (5,3 % versus 2,4 %). Über ähnliche Ergebnisse berichteten van Kasteren et al. [32] in einer multizentrischen Studie mit knapp 2000 primären TEPs. Die Ergebnisse der vorliegenden Umfrage bestätigen die Einhaltung dieser wichtigen, evidenzbasierten Empfehlung in der Primärendoprothetik.

In der klinischen Praxis besteht häufig eine Unsicherheit hinsichtlich des Einflusses einer präoperativen Antibiotikagabe auf die Aussagekraft intraoperativ gewonnener mikrobiologischer Proben. Dies spiegelt sich auch in den vorliegenden Umfrageergebnissen wider: 16 % der befragten Kliniken geben die PAP standardmäßig bei aseptischen Revisionseingriffen nach erfolgter intraoperativer Probeentnahme (also deutlich nach Hautschnitt). Durch diese Praxis wird auf eine wirksame PAP bei einem Eingriff mit nachweislich erhöhtem Infektionsrisiko verzichtet. Obwohl die Datenlage zur Beeinflussung der mikrobiologischen Diagnostik durch die präoperative Antibiotikagabe insgesamt als begrenzt einzustufen ist, zeigen verfügbare Studien, dass eine vor Hautschnitt verabreichte Einmalgabe die mikrobiologischen Kulturergebnisse nicht verfälscht. Tetrault et al. [30] konnten in einer RCT mit kulturpositiven PJI keinen Unterschied in den mikrobiologischen Ergebnissen zwischen präoperativer und intraoperativer Antibiotikagabe feststellen. Zum gleichen Ergebnis gelangten auch Burnett et al. [11] in ihrer prospektiven Untersuchung zu kulturpositiven PJI. Auf Grundlage der vorhandenen Evidenz empfehlen die Autoren dieser Arbeit, die PAP in allen Fällen (auch in Revisions- und Infektionskonstellationen) konsequent vor dem Hautschnitt zu verabreichen* („moderate Evidenzgüte“ *bzw.* „Expertenmeinung“)*.

### Dauer der PAP

Im internationalen Konsens und auf Basis der S3-Leitlinienempfehlung soll die PAP i. d. R. mit dem Operationsende abgeschlossen werden. Nur in ausgewählten Situationen soll sie für eine Dauer von bis zu 24 Stunden fortgeführt werden [4, 8].

Insbesondere in der Revisionsendoprothetik ist die Evidenzlage zur optimalen Dauer der PAP heterogen und unzureichend. Die Umfrageergebnisse verdeutlichen dies eindrucksvoll: Jede dritte Klinik führt die Antibiotikaprophylaxe nach Hautnaht routinemäßig fort und jede zweite Klinik stuft gleichzeitig die Evidenzlage in der Revisionsendoprothetik als gering bis mäßig ein. Diese Einschätzung wird auch durch eine systematische Übersichtsarbeit von Siddiqi et al. [27] sowie von einem Expertengremium (Proceedings of the International Consensus on Orthopedic Infections [1]) geteilt. Daraus wird geschlussfolgert, dass eine evidenzbasierte Empfehlung zur optimalen Dauer der PAP in der Revisionsendoprothetik zum aktuellen Zeitpunkt nicht möglich sei. Es wird aber übereinstimmend angenommen, dass eine Einmalgabe dem deutlich erhöhten Infektionsrisiko von ca. 5 % in der Revisions- und 10–20 % in der Tumorendoprothetik nicht gerecht wird [20, 26, 36]. Zudem gehen PJI mit einer sehr hohen Reinfektionsrate und substanziellen Mortalität einher [23]. Dies verdeutlicht die Notwendigkeit effektiver präventiver Strategien. Eine mögliche Stellschraube könnte dabei die Verlängerung der PAP darstellen. In anderen chirurgischen Disziplinen, wie z. B der Kardiochirurgie, wird bei definierten Eingriffen eine verlängerte PAP für die Dauer von 24 Stunden empfohlen [4]. Unter Berücksichtigung der verfügbaren Evidenz und der Empfehlungen internationaler Fachgremien halten die Autoren dieser Arbeit eine Fortführung der PAP für die Dauer von bis zu 24 Stunden bei klar definierten orthopädisch-unfallchirurgischen „Risikoeingriffen“ für gerechtfertigt. Hierzu zählen Eingriffe mit gleichzeitig erhöhtem Infektionsrisiko und potenziell schwerwiegenden Folgen bei Eintritt einer Infektion. Die Revisions- und Tumorendoprothetik gehören zweifelsfrei zu diesen „Risikoeingriffen“, sodass aus Sicht der Autoren standardisiert eine PAP für bis zu 24 Stunden nach Nahtende erfolgen sollte *(„Expertenmeinung“)*.

Eine über 24 Stunden hinausgehende Antibiotikaprophylaxe besitzt keinen nachgewiesenen Nutzen, erhöht aber signifikant das Risiko für unerwünschte Nebenwirkungen *(„hohe Evidenzgüte“)*. Dieser Umstand konnte im Rahmen einer doppelt verblindeten, multizentrischen RCT (PARITY-trial) mit Implantation von über 600 Tumorprothesen eindrucksvoll gezeigt werden [18]. Die postoperative Gabe von Antibiotika innerhalb von 8 Stunden nach Hautnaht für wahlweise 24 Stunden oder 5 Tage erbrachte keinen Unterschied hinsichtlich der Vermeidung postoperativer Infektionen, es traten jedoch signifikant mehr antibiotikaassoziierte Komplikationen in der 5‑Tage-Gruppe auf. Auch Tay et al. [33] konnten in ihrer Registerstudie mit über 17.000 primären TEPs sogar für Hochrisikopatienten keinen Vorteil für eine PAP, die über 24 Stunden hinaus verabreicht wurde, nachweisen. Der Umstand, dass eine PAP, die nach 24 Stunden fortgesetzt wird, mit dem vermehrten Auftreten unerwünschter Arzneimittelwirkungen (z. B. akutes Nierenversagen (insbesondere bei Vancomycin-Gabe zur PAP) und *Clostridioides-difficile*-Infektionen) assoziiert ist, konnte in mehreren weiteren hochwertigen Studien belegt werden [7, 13]. Dennoch sind weitere prospektive Studien und RCTs dringend notwendig, um die Evidenzlage für zukünftige Leitlinienempfehlungen hinsichtlich der optimalen PAP-Dauer in der Revisions- und Tumorendoprothetik zu stärken.

### Präemptive Therapie

Eine über 24 Stunden hinausgehende PAP ist per definitionem nicht mehr als Prophylaxe, sondern als antibiotische Therapie bzw. präemptive Therapie zu werten [4]. Unter einer präemptiven Therapie verstehen die Autoren dieser Arbeit die Fortsetzung der PAP im Sinne einer empirischen Antibiotikatherapie bis zum Erhalt der mikrobiologischen und histologischen Befunde in Fällen, bei denen intraoperativ ein Infektionsverdacht nicht auszuschließen war. Die Indikationsstellung zur präemptiven Therapie obliegt dem Operateur und muss im Operations- bzw. Arztbericht eindeutig dokumentiert werden. Abhängig von den mikrobiologischen und histologischen Befunden wird die präemptive Antibiotikatherapie beendet oder in eine gezielte antibiotische Behandlung überführt. Aus Sicht der Autoren sollte bei eindeutig negativen Befunden (Mikrobiologie und Histologie) der intraoperativen Gewebeproben die präemptive Therapie beendet und nicht bis zum Ende einer verlängerten Inkubation der letzten mikrobiologischen Proben über 14 Tage abgewartet werden. *(„Expertenmeinung“)*.

### Vorgehen bei anamnestischer Penicillin-Allergie

Laut aktueller Studienlage geben 10–20 % aller hospitalisierten Patienten an, eine Penicillin-Allergie zu haben. Unter dem „Label“ einer Penicillin-Allergie werden jedoch überproportional häufig nichtallergische Reaktionen wie Kopfschmerzen oder gastrointestinale Symptome sowie viral bedingte Exantheme in der Kindheit angegeben. Zahlreiche Studien zeigen, dass der überwiegende Anteil der Patientinnen und Patienten mit anamnestisch angegebener Penicillin-Allergie zum Zeitpunkt einer allergologischen Testung nicht oder nicht mehr allergisch reagiert [9]. Dennoch wird bei diesen Patienten häufig nicht nur die Gabe von Penicillinen, sondern auch von Cephalosporinen aus Furcht vor allergischen Kreuzreaktionen vermieden. Diese Kreuzreaktionen werden i. d. R. durch strukturell ähnliche oder identische Seitenketten der β‑Laktam-Antibiotika verursacht. Cefazolin besitzt keine den Penicillin-Antibiotika strukturell ähnliche Seitenkette, sodass das Risiko von Kreuzreaktionen als sehr gering einzustufen ist [9, 34].

Patienten, die aufgrund der Angabe einer Penicillin-Allergie eine PAP durch ein alternatives Nicht-β-Laktam-Antibiotikum, wie z. B. Vancomycin oder Clindamycin erhalten, weisen ein höheres Risiko für das Auftreten von SSI und PJI auf: In einer retrospektiven Analyse von Kurcz et al. [21] konnte bei der Implantation von nahezu 2500 primären TEPs gezeigt werden, dass sich kein signifikanter Unterschied in der Rate allergischer Reaktionen zwischen Patientinnen und Patienten mit und ohne anamnestisch angegebener Penicillin-Allergie bei Verwendung von Cefazolin (*p* = 0,95) ergab. Hingegen trat bei Anwendung Nicht-Cefazolin-basierter PAP ein signifikant höheres Risiko für PJI auf (2,9 % vs. 5,5 %; *p* = 0,02). Diese Ergebnisse wurden durch die Studie von Wyles et al. [35] mit fast 30.000 primären TEP-Implantationen bestätigt. Im Einklang mit internationalen Leitlinien und der AWMF-S3 Leitlinie sollte die Entscheidung über die Wahl eines zu verabreichenden Antibiotikums bei Patienten, die eine Penicillin-Allergie angeben, risikoadaptiert anhand der klinischen Symptomatik, der Chronologie und des vermuteten Pathomechanismus (IgE-vermittelt, T‑Zell vermittelt) der berichteten mutmaßlichen allergischen Reaktion erfolgen [4].

Bei Patienten mit nichtschwerer Penicillin-Allergie ist der Einsatz von Cefazolin zur PAP möglich und im Konsens mit den Empfehlungen der AWMF-S3 Leitlinie ausdrücklich empfohlen. Bei Hinweisen auf eine schwere Penicillin-Allergie soll auf Clindamycin (unter Beachtung der lokalen Resistenzsituation von *Staphylococcus aureus*) oder Vancomycin (unter Beachtung der im Vergleich zu Cephalosporinen und Clindamycin deutlich verlängerten Infusionsdauer von 60–120 min) ausgewichen werden *(„hohe Evidenzgüte“)*.

## Schlussfolgerungen

Postoperative Infektionen stellen eine der häufigsten Komplikationen in der operativen Orthopädie und Unfallchirurgie dar und sind mit teils erheblichen Konsequenzen für die Betroffenen sowie das Gesundheitssystem verbunden.

Die korrekt durchgeführte perioperative Antibiotikaprophylaxe (PAP) bildet die wichtigste Säule der Infektionsprävention.

**Abb. 3:**
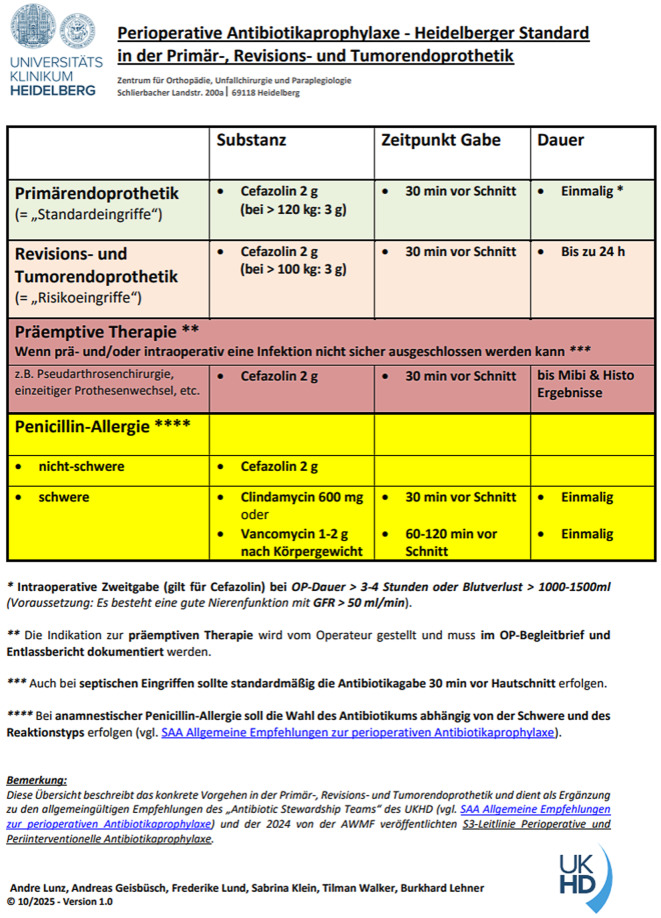
Standardarbeitsanweisung für die perioperative Antibiotikaprophylaxe. *mg* Milligramnn, *g* Gramm, *kg* Kilogramm, *min* Minuten, *h* Stunden, *ml* Milliliter, *Mibi* Mikrobiologie, *Histo* Histologie, *GFR* glomeruläre Filtrationsrate

Auf Grundlage der aktuellen AWMF-S3 Leitlinie und der verfügbaren Evidenz wurde eine konkrete Standardarbeitsanweisung zur PAP in der Primär‑, Revisions- und Tumorendoprothetik erstellt (Abb. 3):

- Cefazolin 2 g i.v. (*„hohe Evidenzgüte“*); gewichtsadaptierte Dosierung: 3 g bei > 100–120 kg Körpergewicht (*„moderate Evidenzgüte“ *bzw. *„Expertenmeinung“*)
- Stringente Applikation ca. 30 Minuten vor Hautschnitt (*„hohe Evidenzgüte“*)
- Verlängerte PAP für bis zu 24 Stunden in der Revisions- und Tumorendoprothetik (*„Expertenmeinung“*)
- Risikoadaptiertes Vorgehen bei anamnestischer Angabe einer Penicillin-Allergie in Abhängigkeit von der Chronologie, der klinischen Symptomatik und des vermuteten Pathomechanismus der Reaktion; Gabe von Cefazolin bei nichtschwerer Penicillin-Allergie (*„hohe Evidenzgüte“*)

## Data Availability

Die in dieser Studie erhobenen Datensätze können auf begründete Anfrage beim Korrespondenzautor angefordert werden.
